# Optimization of Ellagic Acid-Loaded Liposomes Using Box–Behnken Design and the Modulatory Role of Chitosan Molecular Weight on Their Stability, Digestive Release, and Antioxidant Activity

**DOI:** 10.3390/foods15132341

**Published:** 2026-07-02

**Authors:** Wenjia Zhong, Liang He, Liling Wang, Yanbin Wang

**Affiliations:** 1 College of Food and Health, Zhejiang A&F University, Hangzhou 311300, China; shie233@163.com; 2 Zhejiang Academy of Forestry, Hangzhou 310023, China; kite006@126.com (L.H.); echo22239@163.com (L.W.)

**Keywords:** ellagic acid, liposomes, chitosan, stability, antioxidant activity, tyrosinase inhibition

## Abstract

Ellagic acid (EA) possesses various biological activities, including anti-inflammatory, whitening, and antioxidant properties. Its practical application is limited by poor aqueous solubility and susceptibility to degradation. To overcome these limitations, this study prepared EA liposomes using the thin-film hydration–ultrasonication method, followed by surface modification with low-molecular-weight chitosan (LM-CS) and medium-molecular-weight chitosan (MM-CS), yielding EA liposomes modified with LM-CS (EA-L-LC) and MM-CS (EA-L-MC), respectively. The formulation and preparation process were optimized using a Box–Behnken design combined with response surface methodology. Under optimal conditions, the mean particle size (MPS), polydispersity index (PDI), Zeta-potential, and encapsulation efficiency (EE) of the different liposomes (unmodified EA-L, EA-L-LC, and EA-L-MC) were determined. Morphological observation and functional group characterization were conducted via transmission electron microscopy (TEM) and Fourier-transform infrared spectroscopy (FTIR), respectively. The stability of the various liposomes was compared under different environmental conditions, and their stability and the released amount of EA were evaluated during in vitro digestion. The in vitro antioxidant activity and tyrosinase inhibitory effects of the different liposomes were investigated. After process optimization, the encapsulation efficiency of EA liposomes was effectively enhanced following modification with chitosan of different molecular weights. TEM and FTIR results confirmed that EA was effectively encapsulated, and chitosan was successfully coated onto the outer layer of the liposomes. Compared to unmodified EA liposomes (EA-L), the chitosan-modified liposomes (EA-L-LC and EA-L-MC) exhibited enhanced in vitro antioxidant activity and sustained, slow-release tyrosinase inhibitory effects, along with superior stability across multiple conditions. In vitro digestion experiments demonstrated that EA-L-MC and EA-L-LC achieved slower release rates in simulated gastric fluid compared to EA-L, thereby improving the digestive stability of EA.

## 1. Introduction

Ellagic acid (EA), a natural polyphenolic dilactone and a dimeric derivative of gallic acid, is widely distributed in various fruits and nuts, such as strawberries [1], pomegranates [2], and walnuts [3]. In plant tissues, EA primarily exists in a bound form and is released upon the hydrolysis of ellagitannins [4]. It exhibits a broad spectrum of biological functions, including antioxidant, anti-inflammatory, hepatoprotective, skin-whitening, and immunomodulatory activities [5], demonstrating significant potential for application in functional foods, cosmetics, and pharmaceuticals. However, the practical application of EA is limited by two main issues: poor water solubility and poor stability in alkaline environments. These limitations negatively impact its systemic absorption and bioavailability, thereby restricting the development of EA-based products and the expansion of its application scenarios. Consequently, developing effective delivery systems to enhance the solubility and stability of EA remains a pivotal scientific challenge for advancing its functional applications.

Liposomes are closed vesicular structures characterized by a lipid bilayer formed when lipids are dispersed in aqueous solutions. Due to their inherent amphiphilicity, they are capable of simultaneously encapsulating both hydrophobic and hydrophilic compounds [6], making them a widely adopted platform for the construction of bioactive ingredient delivery systems. Although traditional unilamellar liposomes can enhance the solubility of poorly water-soluble components, they often suffer from poor storage stability in low-pH environments and are prone to leakage during gastrointestinal digestion, which ultimately leads to diminished bioavailability [7]. The encapsulation of EA in liposomes for bioactive delivery has been extensively investigated. Heidarian et al. [8] prepared ellagic acid-loaded liposomes with a mean particle size of 125 nm, a PDI of 0.24, a Zeta potential of −12 mV, and an encapsulation efficiency of 23%, which remains relatively low. Modifying liposomes to overcome these drawbacks is essential for improving the delivery efficiency of EA. To address these deficiencies, a common contemporary strategy involves the surface modification of liposomes with biopolymers to improve the EE and structural integrity of the resulting polymer–lipid complexes [9]. Chitosan, a natural cationic polysaccharide enriched with amino and hydroxyl groups along its molecular chain, can be adsorbed onto the surface of negatively charged liposomes through electrostatic interactions, thereby forming a robust protective coating. This chitosan coating not only enhances the structural integrity of liposomes under acidic conditions and enables the controlled release of active compounds, but also exerts antioxidant effects through its amino and hydroxyl groups, which function as hydrogen donors to quench free radicals and interrupt the oxidative chain reaction [10]. Studies have established that the molecular weight of chitosan exerts a profound influence on the performance of liposomal composite systems. Extant studies have mainly focused on the effects of modifying EA liposomes with chitosan of a specific average molecular weight. A systematic investigation into how varying molecular weights of chitosan impact their stability, gastrointestinal release profiles, and antioxidant activities remains notably absent [11].

To enhance the utilization of EA, this study employed the thin-film hydration–ultrasonication method to prepare EA-loaded liposomes, with the preparation process optimized via Box–Behnken response surface methodology following preliminary single-factor experiments. Building upon the optimized formulation, low-molecular-weight and medium-molecular-weight chitosans were utilized for surface modification. The study systematically evaluated the differences between the two modified systems in terms of EE, structural characterization, environmental stability, in vitro digestive release behavior, antioxidant activity, and tyrosinase inhibitory capacity. This study aims to elucidate the influence of chitosan molecular weight on the performance of EA liposomes, thereby providing a robust theoretical foundation and experimental reference for the construction of highly efficient and stable oral delivery systems for EA.

## 2. Materials and Methods

### 2.1. Chemicals and Reagents

Ellagic acid (EA), egg yolk lecithin (EYL), cholesterol, low-molecular-weight chitosan (LM-CS, 200 kDa, degree of deacetylation of 90%), medium-molecular-weight chitosan (MM-CS, 500 kDa, degree of deacetylation of 80%), DPPH (2,2-Diphenyl-1-picrylhydrazyl), ABTS [2,2′-Azino-bis(3-ethylbenzothiazoline-6-sulfonic acid)], and tyrosinase activity assay kit were purchased from Shanghai Acmec Biochemical Technology Co., Ltd. (Shanghai, China). Simulated gastric fluid (SGF) and simulated intestinal fluid (SIF) were obtained from Shanghai Yuanye Bio-Technology Co., Ltd. (Shanghai, China). Methanol, ethanol, sodium chloride, sodium hydroxide, hydrochloric acid, potassium persulfate, phosphoric acid, acetic acid, and tetrahydrofuran (THF) were purchased from Chengdu Chron Chemical Co., Ltd. (Chengdu, China).

### 2.2. HPLC Analysis Conditions

Chromatographic analysis was performed using a HPLC system (Vanquish Core, Thermo Fisher Scientific Inc., Waltham, MA, USA) equipped with a ZORBAX Eclipse XBD-C18 column (5.0 µm, 4.6 mm × 250 mm). The mobile phase consisted of a mixture of methanol and 0.1% aqueous phosphoric acid (55:45, *v*/*v*). The isocratic elution was conducted at a flow rate of 1 mL/min with an injection volume of 30 μL. The effluent was monitored at a detection wavelength of 255 nm.

To establish the quantitative method, an EA stock solution of 0.1 mg/mL was prepared by dissolving 10.0 mg of EA standard in methanol and adjusting the final volume to 100 mL. A series of working solutions with concentrations of 1, 2, 3, 4, 5, 6, 8, 10, and 30 μg/mL was obtained via subsequent dilutions of the stock solution. All solutions were filtered through a 0.45 μm microporous membrane prior to HPLC analysis. Linear regression analysis was performed by plotting the peak area (y) against the mass concentration (x, μg/mL), yielding the calibration equation:

(1) y = 1.7847x − 0.5182 (*R*^2^ = 0.9998)

### 2.3. Preparation of EA-Loaded Liposomes

EA-loaded liposomes were prepared using the thin-film hydration method. Briefly, EA was completely dissolved in 20 mL of THF, followed by the addition of lecithin and cholesterol at a specific mass ratio. The organic solvent was subsequently removed using a rotary evaporator (YRE-5299, Gongyi Yuhua Instrument Co., Ltd., Gongyi, China) under vacuum at 40 °C and 20 rpm until a uniform, transparent lipid film was formed. The resulting film was placed in an oven (BXH-130S, Shanghai Boxun Medical Equipment Co., Ltd., Shanghai, China) at 30 °C overnight to ensure the complete elimination of residual solvent. The lipid film was then hydrated with 10 mL of ultrapure water at 700 rpm. Following complete hydration, the suspension was rapidly cooled and subjected to probe sonication using an ultrasonic cell crusher (JY98-IIIDN, Ningbo Scientz Biotechnology Co., Ltd., Ningbo, China) in an ice bath (operating in cycles of 5 s on and 5 s off). The final product, designated as EA liposomes (EA-L), was obtained and stored for further characterization.

Due to the poor aqueous solubility of free EA, a low-speed centrifugation method was employed to determine the EE of the liposomes. Briefly, a 2 mL aliquot of the liposomal suspension was centrifuged at 2500 rpm and 4 °C for 10 min using a high-speed refrigerated centrifuge (CL31R, Thermo Fisher Scientific Inc., USA) to precipitate the unencapsulated EA. Subsequently, 2 mL of the resulting supernatant was transferred to a 10 mL volumetric flask, where methanol was added to volume to induce membrane disruption (demulsification). The solution was then filtered through a 0.45 μm membrane, and the concentration of encapsulated EA (C_1_) was quantified via HPLC. To determine the total EA concentration (C_0_), another 2 mL of the original liposomal sample was directly disrupted with an appropriate volume of methanol, filtered, and analyzed. The encapsulation efficiency (EE) was calculated according to the following equation:

(2) $$EE\;(\%)=\frac{C_{1}}{C_{0}}\;\times \;100\%$$

where C_1_ is the concentration of encapsulated EA, and C_0_ is the total concentration of EA initially added.

The thin-film hydration method was employed to evaluate the effects of various parameters on the EE of EA liposomes. The experimental variables investigated included the mass ratio of EA to lecithin (1:10, 1:15, 1:20, 1:25, and 1:30), hydration duration (0, 30, 60, 90, 120, and 120 min), and the mass ratio of lecithin to cholesterol (2:1, 3:1, 4:1, 5:1, and 6:1). Additionally, the impact of sonication time (0, 5, 10, 15, and 20 min), hydration temperature (30, 40, 50, 60, and 70 °C), and ultrasonic power intensity (90, 120, 150, 180, and 210 W) were systematically examined.

Based on the results of the preliminary single-factor investigations, the mass ratio of EA to lecithin (1:15, 1:20, and 1:30), hydration duration (30, 60, and 90 min), and the mass ratio of lecithin to cholesterol (4:1, 5:1, and 6:1) were identified as the key independent variables. A Response Surface Methodology (RSM) was subsequently implemented to optimize the extraction process and elucidate the interactive effects of these parameters, ultimately establishing the optimal formulation conditions.

### 2.4. Preparation of Chitosan-Modified EA Liposomes

Low-molecular-weight and medium-molecular-weight chitosan solutions (LM-CS and MM-CS) were prepared following the methodology described by Mork et al. [12]. Briefly, 0.2 g of each chitosan variant was accurately weighed and dissolved in 100 mL of 1% (*v*/*v*) glacial acetic acid. The pH was adjusted to 3.5, and the mixtures were stirred vigorously before being allowed to equilibrate for 48 h. Subsequently, the solutions were filtered under reduced pressure to yield a final chitosan concentration of 0.2% (*w*/*v*). To fabricate the modified liposomes, equal volumes of the chitosan solution and the pre-formed liposomal suspension were utilized. The liposomal suspension was added dropwise into the chitosan solution at ambient temperature under constant magnetic stirring at 700 rpm. Upon completion of the addition, the stirring speed was increased to 1000 rpm for 1 h to ensure uniform coating. The resulting low-molecular-weight chitosan (LM-CS)-modified and medium-molecular-weight chitosan (MM-CS)-modified EA liposomes (EA-L-LC and EA-L-MC, respectively) were stored at 4 °C and protected from light for further use.

### 2.5. Characterization of Mean Particle Size (MPS), Polydispersity Index (PDI), and Zeta-Potential

Three key indicators of blank liposomes (L), EA-L, EA-L-LC, and EA-L-MC were determined by using a Zetasizer (Zetasizer Lab, Malvern Panalytical Ltd., Malvern, UK), including the MPS, PDI and zeta-potential. Prior to analysis, all samples were diluted tenfold with distilled water to mitigate the interference caused by multiple light scatterings, thereby ensuring the accuracy of the measurements.

### 2.6. Transmission Electron Microscopy (TEM)

The morphology of various liposomes was characterized via TEM (Tecnai G2 F30, Thermo Fisher Scientific Inc., USA). The diluted samples were placed on a formvar-coated copper grid and allowed to equilibrate until dry. Subsequently, the specimens were negatively stained by applying a 2% (*w*/*v*) phosphotungstic acid solution. Any excess stain was carefully removed using filter paper, and the grids were air-dried at ambient temperature prior to TEM observation. The structural integrity and surface features of the liposomes were then systematically examined.

### 2.7. Fourier Transform Infrared Spectroscopy (FTIR)

The chemical structures of EA, low- and medium-molecular-weight chitosan, lecithins, and the formulated nanoparticles (L, EA-L, EA-L-LC, and EA-L-MC) were analyzed using FTIR spectroscopy (Nicolet i S50 FT-IR, Thermo Fisher Scientific Inc., USA). Spectra were recorded from 4000 to 400 cm^−1^ at a resolution of 4 cm^−1^, with 32 scans accumulated per sample to improve data quality.

### 2.8. Storage Stability

To evaluate storage stability, L, EA-L, EA-L-LC, and EA-L-MC were stored at 4 °C in the absence of light for a duration of 30 days. The MPS, PDI, Zeta-potential, and retention were measured at specific time points (0, 7, 15, and 30 days). The concentration of EA was determined following the method in Section 2.5, and the retention (R) was calculated using the following equation:

(3) $$R\;(\%)=\frac{\;C_{res}}{C_{init}}\;\times \;100\%$$

where C_init_ is the concentration of ellagic acid encapsulated in the liposomes at the beginning of the experiment, and C_res_ is the concentration of ellagic acid still encapsulated in the liposomes at the end of the experiment.

### 2.9. pH Stability

To evaluate pH stability, the L, EA-L, EA-L-LC, and EA-L-MC suspensions were adjusted to pH 2.0, 4.0, 6.0, 8.0, and 10.0 using HCl or NaOH. The samples were stored at 4 °C, protected from light, for 24 h to allow for structural equilibration. Following the incubation period, the MPS and Zeta potential were measured to characterize the influence of pH on the physicochemical properties and colloidal stability of the formulations.

### 2.10. NaCl Stability

To evaluate ionic stability, the L, EA-L, EA-L-LC, and EA-L-MC suspensions were mixed with an equal volume of NaCl solutions at concentrations of 20, 40, 60, and 80 mg/mL. The samples were stored at 4 °C, protected from light, for 24 h to allow for structural equilibration. Following the incubation period, the MPS and Zeta potential were measured to characterize the influence of NaCl on the physicochemical properties and colloidal stability of the formulations.

### 2.11. In Vitro Simulated Digestion

The compositions of simulated gastric fluid (SGF) and simulated intestinal fluid (SIF) are presented in Table 1. The gastrointestinal stability and release behavior of the liposomal formulations were evaluated using a sequential in vitro digestion model. A 20 mL volume of the prepared liposome suspension was mixed with an equal volume of SGF. The pH of the mixture was adjusted to 1.5, followed by incubation in a shaking incubator (HZ-2111K-B, Changzhou Jebsen Instrument Co., Ltd., Changzhou, China) at 37 °C and 120 rpm for 2 h. Upon completion of the gastric phase, 20 mL of the resulting chyme-like mixture was collected and neutralized with 20 mL of SIF. The pH was subsequently adjusted to 6.8, and the incubation continued under the same conditions (37 °C, 120 rpm) for an additional 4 h. Throughout the SGF and SIF digestion processes, samples were taken every 30 min to measure the release of EA. The EA concentration was determined according to the method in Section 2.5, and the cumulative released amount of EA (ER) was calculated using the following equation:

(4) $$ER\;(\%)=\frac{C_{init}\;{-\;C}_{res}}{C_{init}}\;\times \;100\%$$

where C_init_ denotes the initial concentration of EA in the solution at the onset of the experiment, and C_res_ represents the residual concentration of EA remaining in the solution upon the conclusion of the experiment.

### 2.12. In Vitro Antioxidant Activity

#### 2.12.1. DPPH Radical Scavenging Activity

A 0.1 mM DPPH solution was prepared in anhydrous ethanol, while free EA and the various nanoparticle samples were diluted to a final concentration of 25 μg/mL. Equal volumes of the sample solution and the DPPH solution were thoroughly mixed and incubated at room temperature in the dark for 30 min. The absorbance of the resulting mixture was measured at a wavelength of 517 nm using a microplate reader (Varioskan Flash, Thermo Fisher Scientific Inc., USA). Ascorbic acid (AA) at the same concentration was used as a positive control following the identical procedure. The DPPH radical scavenging activity (SA) was calculated using the following equation:

(5) $${SA}_{DPPH}\;(\%)=(1-\frac{\;A_{sample}\;-\;A_{control}}{A_{blank}})\;\times \;100\%$$

where A_sample_ represents the absorbance of the sample mixed with the DPPH solution; A_control_ is the absorbance of the sample mixed with anhydrous ethanol (without DPPH); and A_blank_ is the absorbance of the DPPH solution mixed with anhydrous ethanol (without sample).

#### 2.12.2. ABTS Radical Scavenging Activity

The ABTS·+ stock solution was generated by mixing 7 mM ABTS and 2.45 mM potassium persulfate (K_2_S_2_O_8_) in equal volumes, followed by incubation in the dark at room temperature for 12–16 h. Before use, the solution was diluted with 10 mM phosphate-buffered saline (PBS) to achieve a target absorbance of 0.70 ± 0.02 at 734 nm, yielding the ABTS working solution. A 3.6 mL volume of the ABTS working solution was mixed with 0.4 mL of the sample solution, and the absorbance was recorded at 734 nm. Ascorbic acid (AA) at the same concentration was used as the positive control and was processed under identical conditions. The ABTS radical scavenging activity (SA) was calculated according to the following equation:

(6) $${SA}_{ABTS}\;(\%)=(1-\frac{\;A_{sample}\;-\;A_{control}}{A_{blank}})\;\times \;100\%$$

where A_sample_ represents the absorbance of the sample mixed with the ABTS solution; A_control_ is the absorbance of the sample mixed with PBS (without ABTS); and A_blank_ is the absorbance of the ABTS solution mixed with PBS (without sample).

### 2.13. In Vitro Inhibition of Tyrosinase Activity

The inhibitory effect on tyrosinase was determined based on the enzymatic oxidation of L-DOPA to dopaquinone, which subsequently reacts with MBTH (3-methyl-2-benzothiazolinone hydrazone) to form a chromogenic pink–red complex with a characteristic absorption peak at 505 nm. Free EA and the various liposomal samples were diluted to a range of concentrations. A 50 μL volume of tyrosinase solution was mixed with 50 μL of the sample solution and 900 μL of L-DOPA. The reaction mixture was conducted at 37 °C for 40 min, after which the absorbance was recorded at 505 nm to evaluate the enzymatic activity. The tyrosinase inhibition activity (TI) was calculated according to the following equation:

(7) $$TI\;(\%)=(1-\frac{A_{2}\;-{\;A}_{3}}{A_{1}\;-\;A_{0}})\;\times \;100\%$$

where A_0_ represents the absorbance of the blank group consisting of PBS and L-DOPA; A_1_ is the absorbance of the control group containing the enzyme, PBS, and L-DOPA; A_2_ denotes the absorbance of the test group containing the enzyme, the sample, and L-DOPA; and A_3_ refers to the background absorbance of the sample group consisting of the sample, PBS, and L-DOPA.

To investigate the enzymatic kinetics of tyrosinase in vitro, 50 μL of tyrosinase solution was mixed with 50 μL of sample solution (EA concentration of 400 μg/mL) and 900 μL of L-DOPA. The reaction was conducted at 37 °C, and the absorbance was measured at 505 nm after incubation periods of 5, 10, 15, 20, 25, 30, 35, and 40 min. The tyrosinase inhibition activity of different samples at each time point was subsequently calculated using Equation (7).

Nonlinear regression analysis of the tyrosinase inhibition data was performed using Origin software to construct a dose–response regression equation between concentration and inhibition activity. The half-maximal inhibitory concentration (IC_50_) was then calculated by solving the regression equation for the sample concentration corresponding to 50% inhibition.

### 2.14. Statistical Analysis

All experiments were conducted in triplicate independently, and the results are presented as the mean ± SD. Data organization and preliminary calculations were performed using WPS Office 2019. Statistical analyses were carried out using SPSS 27.0. One-way ANOVA with Tukey’s post hoc test was used for multiple group comparisons. The RSM design and subsequent optimization analysis were carried out using Design-Expert 13, while all graphical illustrations were rendered using Origin 2024.

## 3. Results and Discussion

### 3.1. Single-Factor Experimental

#### 3.1.1. Effect of the Mass Ratio of EA to Lecithin on EE

**Figure 1:**
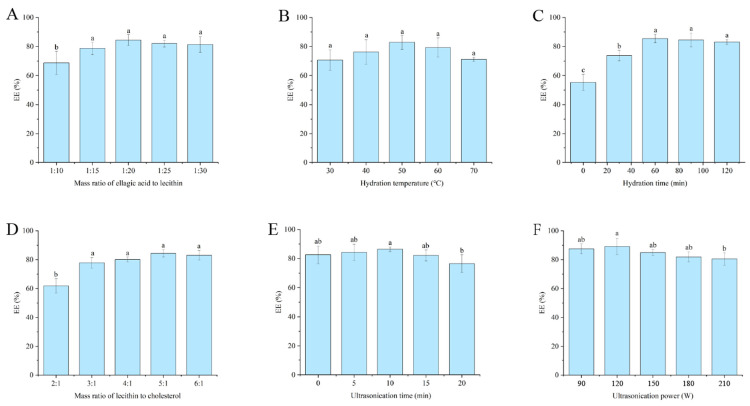
Results of single-factor experiments on preparation of composite liposomes. Effect of the mass ratio of EA to lecithin (**A**), hydration temperature (**B**), hydration time (**C**), mass ratio of lecithin to cholesterol (**D**), ultrasonication time (**E**), and ultrasonication power (**F**) on the EE of the liposomes (*n* = 3). Different small letters indicate significant differences (*p* < 0.05).

As illustrated in Figure 1A, the EE reached its peak when the mass ratio of EA to lecithin was 1:20. This phenomenon could be attributed to the fact that at excessively high EA proportions, the core material content exceeds the saturated loading capacity of the liposomes. Such oversaturation tends to compromise the structural stability and membrane integrity of the vesicles [13], leading to the leakage of EA. Increasing the phospholipid content within a specific range facilitates the effective entrapment of EA molecules [14,15]. An inordinate increase in lecithin concentration results in elevated system viscosity, which may impede the diffusion of EA into the liposomal bilayers, thereby leading to a subsequent decline in EE [16].

#### 3.1.2. Effect of Hydration Temperature on EE

As illustrated in Figure 1B, the EE of EA liposomes increased initially but then declined with rising hydration temperatures. This phenomenon may be attributed to the specific phase state of the phospholipids: at lower hydration temperatures, the phospholipids remain in a rigid gel phase as the temperature has not yet reached the phase transition temperature, which hinders effective EA entrapment. Conversely, excessively high hydration temperatures significantly increase the fluidity of the lipid bilayer and may break hydrogen bonds, leading to EA leakage [17]. Consequently, 50 °C was identified as the optimal hydration temperature for the formulation.

#### 3.1.3. Effect of Hydration Time on EE

As illustrated in Figure 1C, the EE of EA liposomes increased rapidly with the extension of hydration time up to 60 min. This observation could be attributed to the incomplete hydration process at shorter times, which results in a non-uniform distribution of liposomes and the formation of large molecular aggregates or precipitates. Conversely, an excessively prolonged hydration duration may lead to the oxidative degradation of phospholipid molecules, weakening the lipid bilayer and causing EA to leak out [18], which ultimately results in a decline in EE.

#### 3.1.4. Effect of Lecithin-to-Cholesterol Mass Ratio on EE

As illustrated in Figure 1D, the EE of EA liposomes exhibited an upward trend with the increasing mass ratio of lecithin to cholesterol. This phenomenon may be attributed to the incorporation of an appropriate amount of cholesterol, which modulates the fluidity of the lipid bilayer and subsequently enhances the entrapment capacity [19]. Too much cholesterol may disrupt the structural stability of the lipid bilayer, leading to a reduction in the EE [20].

#### 3.1.5. Effect of Ultrasonication Time on EE

As illustrated in Figure 1E, the duration of ultrasonication exerted a relatively minor influence on the EE, with the maximum value observed at 10 min. Excessively prolonged sonication may trigger intense cavitation effects, which could compromise the structural integrity of the liposomes and induce the leakage of EA, thereby leading to a subsequent decline in EE [21]. Consequently, 10 min was established as the optimal ultrasonication time for this study.

#### 3.1.6. Effect of Ultrasonication Power on EE

As illustrated in Figure 1F, the EE of the liposomes exhibited an initial marginal increase followed by a significant decline as the ultrasonication power intensified. This trend may be attributed to the insufficient dispersion and the tendency of liposomes to form molecular aggregates at lower power levels, which limits the effective contact area between phospholipid and EA molecules, thereby reducing the EE. In contrast, excessively high ultrasonication power generates intense shear forces that can rupture the liposomal membranes, leading to the leakage of the encapsulated cargo. This observation is consistent with the findings reported by Guner et al. [22]. Based on these results, 120 W was identified as the optimal ultrasonication power for the preparation process.

### 3.2. Results and Analysis of RSM Experiments

#### 3.2.1. Regression Model Design and ANOVA

Building upon the single-factor experimental results, the hydration temperature, ultrasonication time, and ultrasonication power were fixed at 50 °C, 10 min, and 120 W, respectively. Three independent variables that significantly influenced the EE, namely the mass ratio of drug to lecithin (A), hydration time (B), and the mass ratio of lecithin to cholesterol (C), were selected for further optimization. A three-factor, three-level RSM based on the Box–Behnken design (BBD) was employed, with the EE of EA liposomes serving as the response value. The experimental design and corresponding results are summarized in Table 2. Multiple regression fitting analysis via Design-Expert 13.0, a second-order polynomial regression equation for the EE (Y), was established as follows:

(8) Y = 85.24 + 2.64A + 1.35B + 0.7388C − 0.0700AB − 1.05AC − 0.5225BC − 3.73A^2^ − 2.84B^2^ − 3.07C^2^

As evidenced by the ANOVA results presented in Table 3, the regression model yielded a *p* < 0.0001, indicating that the model is highly significant. The lack-of-fit term was non-significant (*p* > 0.05), suggesting that the model fits the experimental data well with minimal experimental error and possesses substantial statistical significance. The coefficient of determination *R*^2^ was 0.9896, and the adjusted coefficient $R_{adj}^{2}$ was 0.9763. Both values are close to 1, further demonstrating the high reliability and precision of the model.

Regarding the significance tests of the regression coefficients, the linear terms (A, B, and C), the quadratic terms (A^2^, B^2^, and C^2^), and the interaction term (AC) all exerted highly significant effects on the EE (*p* < 0.01). The interaction terms (AB and BC) showed no significant impact (*p* > 0.05). Based on the F-values, the relative influence of the three factors on the EE of EA liposomes followed the order: drug-to-lecithin ratio (A) > hydration time (B) > lecithin-to-cholesterol ratio (C). As the predominant factor, the drug-to-lecithin ratio directly dictates the loading capacity and entrapment efficiency of the phospholipid bilayer for EA molecules. An excessively high drug-to-lecithin ratio exceeds the liposomal carrying capacity, leading to drug precipitation; an insufficiently low ratio results in an inadequate concentration gradient, which hinders the diffusion and entrapment of the drug into the liposomes, thereby reducing the drug loading.

**Figure 2:**
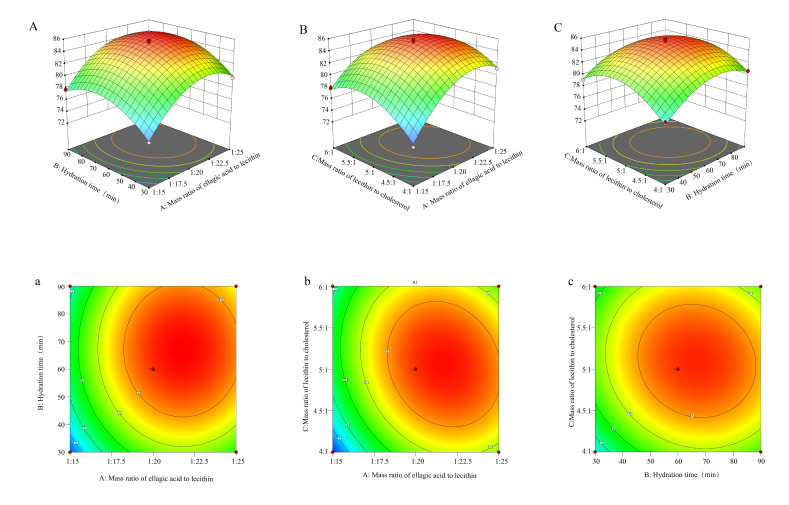
Response 3D and 2D surface plots. (**A**) 3D surface illustrating the interaction between the mass ratio of ellagic acid to lecithin and hydration time; (**B**) 3D surface illustrating the interaction between the mass ratio of ellagic acid to lecithin and the mass ratio of lecithin to cholesterol; (**C**) 3D surface illustrating the interaction between the mass ratio of lecithin to cholesterol and hydration time; (**a**) Contour plot of the mass ratio of ellagic acid to lecithin and hydration time; (**b**) Contour plot of the mass ratio of ellagic acid to lecithin and the mass ratio of lecithin to cholesterol; (**c**) Contour plot of mass ratio of lecithin to cholesterol and hydration time.

The steepness of the response surfaces provides a visual representation of the interaction intensity between variables and their respective impacts on the EE of EA liposomes. A steeper surface with rapid color transitions signifies a more pronounced interaction and a greater influence on the response, whereas a gentler slope indicates a diminished effect. As depicted in Figure 2, the response surface for the AC interaction (drug-to-lecithin ratio and lecithin-to-cholesterol ratio) is notably steep with elliptical contour lines, suggesting a significant synergistic effect between these two variables, which aligns with the ANOVA results.

At a lower drug-to-lecithin ratio (1:15), variations in the lecithin-to-cholesterol ratio exerted a negligible influence on the EE. As the drug-to-lecithin ratio increased within the range of 1:15 to 1:25, the EE exhibited a parabolic trend—initially increasing and subsequently declining—in response to the lecithin-to-cholesterol ratio. This phenomenon elucidates the critical roles of phospholipids and cholesterol in liposome formation: while phospholipids constitute the backbone of the lipid bilayer, cholesterol modulates the stability and encapsulation performance by regulating membrane fluidity [23]. At lower drug-to-lecithin ratios, the abundance of phospholipid molecules facilitates the entrapment of EA. Under these conditions, the stabilizing effect of cholesterol is relatively limited. At higher drug-to-lecithin ratios, the increased cargo loading poses a greater resistance to stable vesicle formation. An appropriate increment of cholesterol can effectively increase membrane thickness and enhance structural flexibility, thereby minimizing drug leakage. Nevertheless, excessive cholesterol disrupts the ordered arrangement of the bilayer, leading to a reduction in EE. The synergy between the drug-to-lecithin ratio and the lecithin-to-cholesterol ratio reflects a dynamic equilibrium between drug loading and membrane stability. In comparison, the response surfaces for the AB (drug-to-lecithin ratio and hydration time) and BC (hydration time and lecithin-to-cholesterol ratio) interactions appeared relatively flat, indicating that their interactions are not statistically significant. This suggests that the influence of hydration time on the EE operates independently of the other factors.

#### 3.2.2. Optimal Conditions and Experimental Verification

Based on the single-factor and RSM experiments, the predicted optimal preparation conditions for EA liposomes were determined as follows: ultrasonication power of 120 W, ultrasonication time of 10 min, hydration temperature of 50 °C, drug-to-lecithin ratio of 1:21.727, hydration time of 66.873 min, and a lecithin-to-cholesterol ratio of 5.041:1. Considering the feasibility of practical operation, the parameters were slightly adjusted to a drug-to-lecithin ratio of 1:22, a hydration time of 67 min, and a lecithin-to-cholesterol ratio of 5:1. Under these optimized conditions, triplicate validation experiments yielded an actual EE of 85.93 ± 0.57%, which showed a minimal relative error of only 0.08% compared to the predicted value of 85.86%. Such consistency indicates that the response surface model is highly credible and that the optimized process is stable and reproducible for the preparation of EA liposomes.

### 3.3. EE, Average Particle Size, and Zeta-Potential of Different Liposomes

The results pertaining to the MPS, PDI, Zeta-potential, and EE of the optimized EA-loaded liposomes (EA-L), blank liposomes (L), and EA liposomes coated with LM-CS (EA-L-LC) and MM-CS (EA-L-MC) are summarized in Table 4. Upon the encapsulation of EA, the MPS of the liposomes increased from 76.10 ± 3.11 nm (L) to 113.73 ± 7.90 nm, signifying the successful entrapment of EA within the liposomal architecture. The EA-L formulation exhibited an MPS of approximately 100 nm with a PDI of less than 0.30 and a Zeta-potential of −35.87 ± 0.21 mV. These findings are consistent with the results reported by Heidarian et al. [8], collectively demonstrating that EA was effectively loaded into the liposomes while maintaining excellent dispersibility and colloidal stability.

Following surface modification with chitosan, the MPS of EA-L-MC and EA-L-LC increased to 250.83 ± 7.02 nm and 219.27 ± 1.80 nm, respectively, signifying the successful deposition of chitosan onto the liposomal surface. The MPS of liposomes modified with LM-CS (EA-L-LC) was significantly smaller than that of those modified with MM-CS (EA-L-MC) (*p* < 0.05), an observation consistent with the findings of Mork et al. [12] regarding epicatechin encapsulation systems. This disparity in particle size may be attributed to the distinct chain conformations of chitosan with different molecular weights. Medium-molecular-weight chitosan possesses more extended molecular chains in solution, resulting in a thicker coating layer upon adsorption [24]. The shorter molecular chains of LM-CS facilitate the formation of a more compact and uniform coating, thereby limiting the increment in particle size. The PDI values for all samples ranged from 0.10 to 0.13, well below the threshold of 0.30, indicating a narrow size distribution and superior dispersibility of the prepared liposomes. A slight decrease in PDI was observed following chitosan modification. This improvement in homogeneity could be ascribed to the increased absolute value of the surface charge upon chitosan addition, which enhances the electrostatic repulsion between nanoparticles and further inhibits liposomal aggregation [25].

The Zeta-potential of blank liposomes (L) was measured at −47.07 ± 0.85 mV, with the negative charge originating from the ionization of phosphate groups in the phospholipid molecules. Upon the encapsulation of EA, the absolute Zeta-potential of EA-L decreased to −35.87 ± 0.21 mV. This reduction may be attributed to the formation of hydrogen bonds between the hydroxyl groups of EA and the polar headgroups of the phospholipids, which partially neutralizes the surface charge [26]. Following the addition of cationic chitosan, the Zeta-potentials of EA-L-MC and EA-L-LC shifted from negative to positive values. This charge reversal serves as key evidence for the successful coating of chitosan onto the liposomal surface [27]. The positively charged chitosan molecules are adsorbed onto the negatively charged liposomal templates through electrostatic interactions, imparting a high positive charge density to the composite particles. The absolute Zeta-potential increased significantly from 35.87 mV to 66.23 mV and 63.87 mV, respectively. These results indicate that the chitosan coating not only alters the nature of the surface charge but also substantially enhances the electrostatic repulsion between particles, thereby contributing to the long-term colloidal stability of the system.

No statistically significant difference was observed in the Zeta-potentials of EA-L-MC and EA-L-LC (*p* > 0.05), indicating that the surface charge densities provided by the two types of chitosan were essentially comparable under the preparation condition of pH 3.5. This result stands in stark contrast to the trend observed in particle size, where EA-L-LC exhibited a significantly smaller diameter. Such a discrepancy suggests that the influence of chitosan molecular weight on particle size is mediated primarily through the thickness of the coating layer rather than the charge density. Furthermore, the EE increased significantly following the addition of chitosan (*p* < 0.05). This enhancement may be attributed to the robust mucoadhesive properties of the outer chitosan layer [28], which can interact with the hydroxyl groups of free EA via hydrogen bonding to form stable complexes [29,30], thereby minimizing the loss of the encapsulated cargo.

### 3.4. TEM Analysis

**Figure 3:**
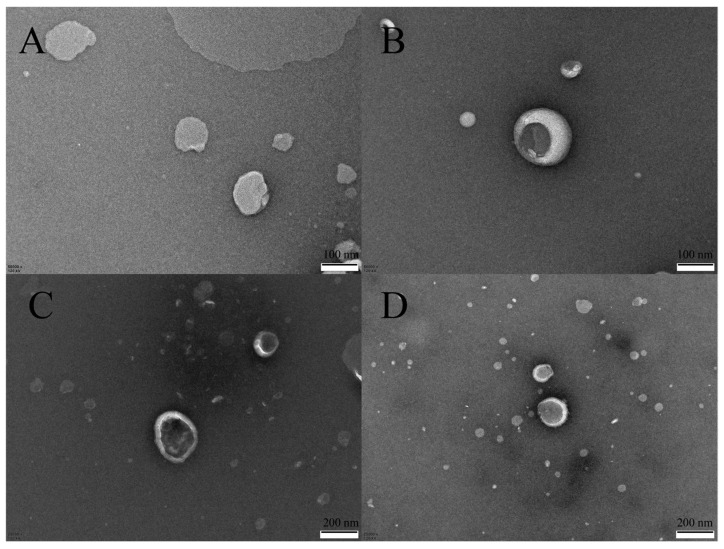
TEM images of different liposomes. L (**A**), EA-L (**B**), EA-L-MC (**C**), EA-L-LC (**D**).

To evaluate the microscopic architecture of the various formulations, TEM was employed to observe the morphology of the nanoparticles. The L, EA-L, EA-L-LC and EA-L-MC all exhibited uniform dispersion and regular geometric shapes. Figure 3 presents the micrographs of L and EA-L at 100 nm magnification, as well as EA-L-MC and EA-L-LC at 200 nm magnification. In accordance with the study by Hu et al. [31], Figure 3A displays typical spherical vesicular structures characteristic of blank liposomes with excellent monodispersity. In Figure 3B, EA-L similarly maintained a spherical morphology, where the lipid bilayer structure is clearly discernible; the observed increase in particle size compared to L signifies the successful entrapment of EA. Occasional smaller vesicles in the field of view may represent blank liposomes that did not encapsulate the drug. Figure 3C,D reveal that the underlying liposomal morphology remained largely unaltered, with the lipid bilayers still distinctly visible. The small, light-colored vesicles observed may be attributed to minor aggregates of chitosan and niosomes. Notably, the particle sizes of both chitosan-coated formulations were larger than those of EA-L, further confirming the successful deposition of chitosan onto the outer layer of the liposomes. The maintenance of a well-defined spherical shape suggests that the chitosan coating process exerted no adverse effects on the structural integrity of the liposomes.

### 3.5. FTIR Analysis

**Figure 4:**
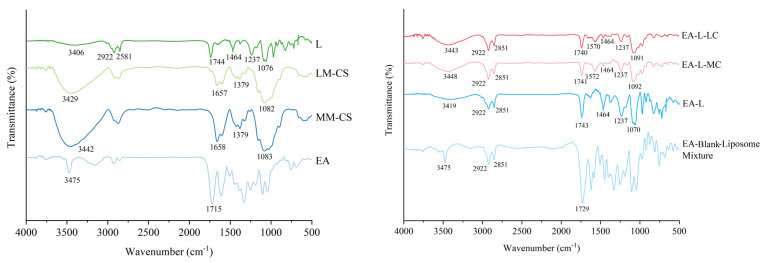
FTIR spectra of pure ingredients (EA, LM-CS, MM-CS), liposome (L, EA-L, EA-L-LC, EA-L-MC) and EA–blank–liposome mixture.

FTIR spectroscopy was employed to characterize the structural features of the pure components (EA, LM-CS, and MM-CS), the various nanoliposomes (L, EA-L, EA-L-LC, and EA-L-MC), and a physical mixture of EA and blank liposomes (EA–blank–liposome mixture). The resulting spectra are illustrated in Figure 4.

The FTIR spectrum of EA exhibited a prominent characteristic peak at 3475 cm^−1^, corresponding to O-H stretching vibrations. Additionally, several subtle absorption bands were observed within the 700–1750 cm^−1^ range, attributed to the aromatic ring skeleton and various functional groups, such as C-O and C-C. The infrared profiles of the two types of chitosan were highly comparable, featuring O-H stretching vibration peaks at 3442 cm^−1^ (MM-CS) and 3429 cm^−1^ (LM-CS), respectively. Both variants displayed a C=O stretching vibration band near 1658 cm^−1^ (MM-CS) and 1657 cm^−1^ (LM-CS), alongside a distinct C-H bending vibration peak at approximately 1379 cm^−1^. This latter feature is characteristic of the amide II band, indicating a substantial presence of residual acetyl groups in both chitosan types [32]. The peaks detected at 1083 cm^−1^ (MM-CS) and 1082 cm^−1^ (LM-CS) are associated with the symmetric and asymmetric stretching vibrations of the PO_2_^−^ groups.

The FTIR spectrum of blank liposomes (L) displayed several distinct characteristic peaks: 1744 cm^−1^ (C=O symmetric stretching vibration), 2922 cm^−1^ and 2851 cm^−1^ (asymmetric and symmetric stretching vibrations of CH_2_ in the acyl chains), 1076 cm^−1^ and 1237 cm^−1^ (symmetric and asymmetric stretching vibrations of PO_2_^−^ groups), and 1464 cm^−1^ (asymmetric stretching vibration of CH_3_) [33,34,35].

No characteristic peak of EA was detected in EA-L, while both EA and blank liposome characteristic peaks were present in the physical mixture of EA and liposomes, indicating that EA was effectively encapsulated. Meanwhile, the symmetric stretching vibration of C=O shifted from 1744 cm^−1^ to 1743 cm^−1^, and the symmetric and asymmetric stretching vibration peaks of PO_2_^−^ shifted from 1076 cm^−1^ to 1070 cm^−1^, suggesting the possible involvement of hydrogen bonding and electrostatic interactions during the encapsulation of EA into liposomes. The symmetric and asymmetric stretching vibrations of CH_2_ in the propylene chain did not shift, which may indicate that the encapsulation of EA had no significant effect on the liposome structure [36].

Following surface modification with chitosan, new absorption peaks emerged at 1570–1572 cm^−1^ in the spectra of both EA-L-LC and EA-L-MC. These peaks are assigned to the bending vibrations of the protonated amino groups (-NH_3_^+^) of chitosan, providing direct evidence of the successful adsorption of chitosan onto the liposomal templates. Compared to the EA-L formulation, the C=O stretching vibration peak in the chitosan-modified liposomes shifted from 1743 cm^−1^ to 1740 cm^−1^ (EA-L-LC) and 1741 cm^−1^ (EA-L-MC), respectively. Concurrently, the absorption band at 1070 cm^−1^ underwent a significant bathochromic shift to 1091 cm^−1^ (EA-L-LC) and 1092 cm^−1^ (EA-L-MC). These spectral alterations collectively demonstrate that chitosan was effectively coated onto the liposomal surface. The formation of the composite nanoliposomes may be attributed to the combined effects of hydrogen bonding and electrostatic interactions [37].

### 3.6. Stability Analysis of Different Liposomes

#### 3.6.1. Storage Stability

**Figure 5:**
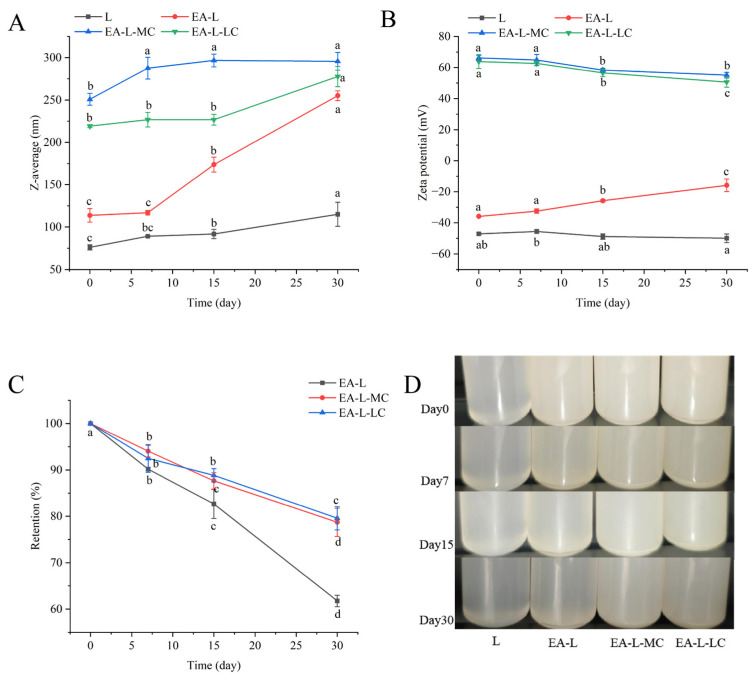
MPS (**A**), Zeta-potential (**B**), and retention (**C**) of L, EA-L, EA-L-MC, EA-L-LC for 30 days (*n* = 3). Appearance of different liposome suspensions after 30 days of storage at 4 °C (**D**). Different small letters indicate significant differences (*p* < 0.05).

To systematically evaluate the impact of chitosan modification on the storage stability of different liposomes, changes in visual appearance, MPS, Zeta-potential and EA retention were monitored over a 30-day storage period. The samples were stored at 4 °C and in the dark, with aliquots withdrawn for analysis at predetermined intervals of 0, 3, 7, 15, and 30 days. The corresponding results are illustrated in Figure 5.

At the end of the 30-day storage period, varying degrees of precipitation were observed in all samples except for the blank liposomes (L), indicating changes in liposomal stability over time. Specifically, the EA-L exhibited the most pronounced sedimentation, whereas the chitosan-modified formulations (EA-L-MC and EA-L-LC) remained relatively homogeneous with minimal precipitation. As illustrated in Figure 5A, the MPS of EA-L increased significantly (*p* < 0.05) from 113.70 ± 7.90 nm to 255.10 ± 5.76 nm, representing a substantial increment of 124.36%. Concurrently, its Zeta-potential rose from −35.87 ± 0.21 mV to −15.93 ± 4.05 mV, a 55.59% increase compared to the initial value. Chitosan-coated liposomes demonstrated superior dimensional stability; the MPS increased from 250.80 ± 7.02 nm (EA-L-MC) and 219.13 ± 1.80 nm (EA-L-LC) to 295.73 ± 10.43 nm and 277.67 ± 11.94 nm, respectively, corresponding to a relatively modest increase of 17.91% and 26.71%. Their Zeta-potentials decreased from 66.23 ± 1.69 mV and 63.87 ± 4.46 mV to 55.07 ± 1.86 mV and 50.6 ± 3.32 mV, representing reductions of 16.85% and 20.78%, respectively. These findings suggest that the chitosan coating intensified the electrostatic repulsion between particles, thereby effectively inhibiting liposomal aggregation during storage [38]. Despite having a larger initial size, EA-L-MC exhibited smaller fluctuations in both particle size and Zeta-potential compared to EA-L-LC after 30 days, signifying its superior stability over the storage duration.

As illustrated in Figure 5C, the retention rates of EA in all liposomal formulations exhibited a progressive decline throughout the 30-day storage period. By the end of the storage duration, the retention of EA-L had dropped to only 61.75%. The chitosan-coated formulations, EA-L-MC and EA-L-LC, maintained significantly higher retention of 78.72% and 79.57%, respectively (*p* < 0.05). These results demonstrate that the application of a chitosan coating effectively enhances the encapsulation stability of the liposomes, which is consistent with the findings reported by Hemmingsen et al. [39].

These findings demonstrate that chitosan modification significantly enhances the storage stability of EA-loaded liposomes, primarily by suppressing particle size growth, maintaining Zeta-potential stability, and improving drug retention. EA-L-MC exhibited slightly superior dimensional stability compared to EA-L-LC. This enhanced stability may be attributed to the formation of a more robust and thicker coating layer by the former [24]. When selecting the optimal chitosan molecular weight for future applications, a strategic balance should be struck between precise particle size control and long-term storage stability based on specific practical requirements.

#### 3.6.2. pH Stability

**Figure 6:**
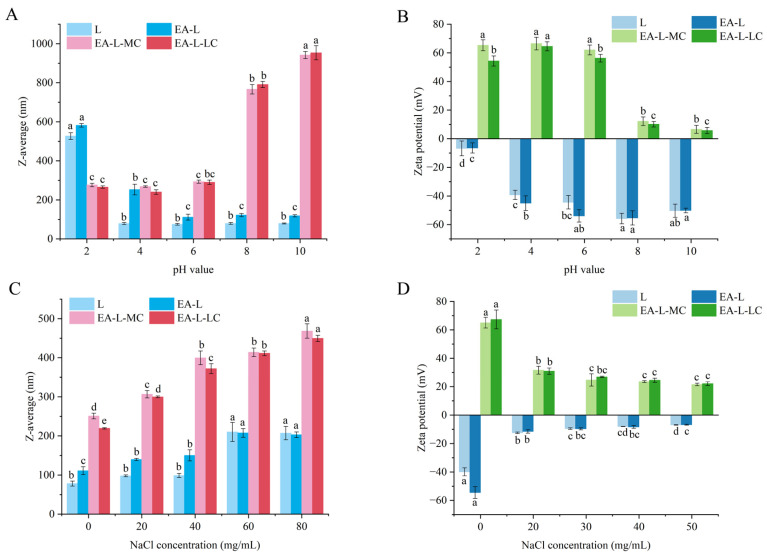
Effect of pH value on the MPS (**A**) and Zeta-potential (**B**) of different liposomes. Effect of NaCl concentration on the MPS (**C**) and Zeta-potential (**D**) of different liposomes (*n* = 3). Different small letters indicate significant differences (*p* < 0.05).

Figure 6A,B illustrate the variations in MPS and Zeta-potential of different liposomal formulations across a range of pH conditions. At pH 2.0, both L and EA-L exhibited a rapid expansion in particle size, reaching 527.03 ± 17.17 nm and 581.63 ± 9.11 nm, respectively. Concurrently, the absolute Zeta-potential values for L and EA-L plummeted to −6.76 ± 5.19 mV and −6.48 ± 3.59 mV, respectively. This phenomenon can be attributed to the reduced surface charge of phospholipids in highly acidic media, which causes the absolute potential to diminish or approach neutrality. The resulting attenuation of electrostatic repulsion triggers inter-liposomal aggregation [40]. These findings collectively indicate that unmodified liposomes possess poor stability under acidic conditions.

The chitosan-coated EA liposomes maintained relative stability in particle size under low pH conditions, with only a marginal increase observed at pH 2.0. This suggests that the chitosan layer acted as a protective barrier on the liposomal surface, effectively mitigating the detrimental effects of acidic environments on structural integrity [9]. Under alkaline conditions, the particle sizes of EA-L-MC and EA-L-LC increased sharply, reaching 766.47 ± 23.93 nm and 791.03 ± 15.36 nm at pH 8.0, respectively, accompanied by visible precipitation and a rapid decline in the absolute Zeta-potential. This phenomenon is likely attributed to the intrinsic properties of the D-glucosamine units within the chitosan structure. When the pH is below the isoelectric point of chitosan, the polymer remains positively charged due to the protonated amino groups (-NH_3_^+^). As the pH increases, the deprotonation of these amino groups leads to a reduction in surface charge density, subsequently triggering liposomal aggregation and size enlargement [41]. The EA-L-LC formulation, coated with LM-CS, exhibited more pronounced fluctuations in particle size compared to the EA-L-MC counterpart, indicating a higher degree of pH sensitivity in the former. In light of the robust stability exhibited by chitosan-coated liposomes in acidic media, and considering the physiological pH gradient characteristic of the human gastrointestinal tract, subsequent simulated digestion assays are warranted to further elucidate their functional performance.

#### 3.6.3. NaCl Stability

As depicted in Figure 6C,D, an increase in salt concentration led to a significant reduction in the absolute Zeta-potential values (*p* < 0.05) for all liposomal formulations, accompanied by fluctuations and an overall upward trend in the MPS. This phenomenon can be attributed to the neutralization of surface charges by cations present in the saline solution, which induces an electrostatic shielding effect. Under the influence of this shielding effect, the surface charge density of the liposomes diminishes, leading to a corresponding decrease in the magnitude of the Zeta-potential. The electrostatic repulsion between the particles is attenuated, triggering liposomal aggregation [42], which manifests as increased particle size and compromised systemic stability. Regardless of chitosan modification, all liposomes exhibited poor stability in high-salinity environments. This indicates that while the chitosan coating may offer a degree of protection, it remains insufficient to maintain the structural integrity and colloidal stability of the system under conditions of high ionic strength.

### 3.7. In Vitro Simulated Gastrointestinal Stability

**Figure 7:**
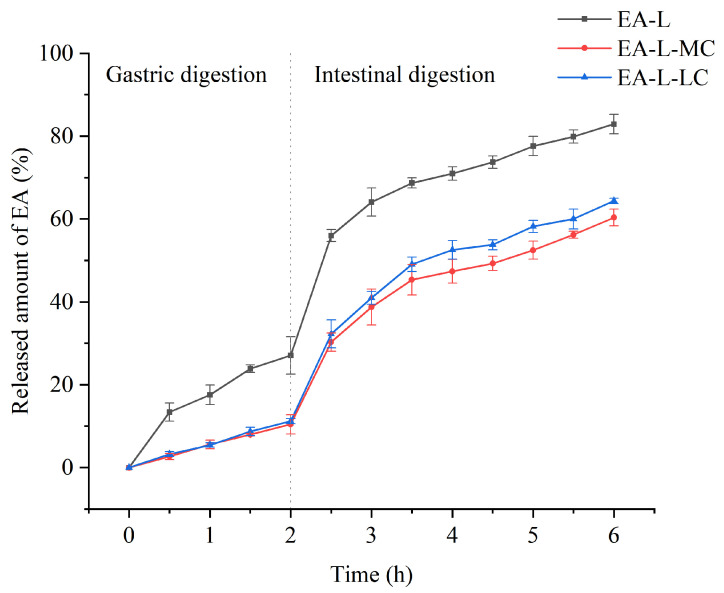
Released amount of EA from liposomes (EA-L, EA-L-MC, EA-L-LC) in SGF digestion for 120 min and in SIF digestion for 240 min (*n* = 3).

The release profiles of the various liposomal formulations were evaluated within a simulated gastrointestinal environment, encompassing 120 min of exposure to simulated gastric fluid (SGF) followed by 240 min in simulated intestinal fluid (SIF). The resulting in vitro digestion kinetics are illustrated in Figure 7.

The results indicated that EA-L exhibited high sensitivity to the simulated digestive environment. By the end of the gastric phase, the cumulative release of EA from EA-L reached 27.06%. In the low-pH environment of SGF, the protonation of phospholipids induces structural alterations in the bilayer, thereby triggering the premature leakage of EA [43]. Upon transitioning into the intestinal environment, the synergistic action of bile salts and pancreatin further compromises the liposomal integrity. Bile salts insert into the lipid bilayer, disrupting the ordered arrangement of lipid molecules, increasing membrane fluidity, and facilitating the formation of mixed micelles [44]. Simultaneously, pancreatic lipase hydrolyzes the ester bonds of phospholipids, leading to the disintegration of the liposomal structure [45].

Upon exposure to the SIF environment, the elevated pH triggers the deprotonation of the amino groups in chitosan, leading to the partial disintegration of the chitosan coating on the liposomal surface [41], thereby modulating the release profile of EA. By the end of the simulated digestion, the cumulative EA release from EA-L reached 82.91%. The released amount of EA for EA-L-MC was reduced by 16.62 percentage points in SGF and 22.57 percentage points in SIF, while that for EA-L-LC decreased by 15.83 percentage points in SGF and 18.55 percentage points in SIF, relative to the uncoated EA-L. These results demonstrate that chitosan encapsulation effectively enhances the membrane rigidity and impermeability of the liposomes [46]. This modification mitigates the premature leakage of EA in the SGF, thereby ensuring that a higher proportion of the encapsulated EA reaches the small intestine for subsequent digestion and absorption. The final released amount of EA of EA-L-LC (64.36%) at the conclusion of digestion was slightly higher than that of EA-L-MC (60.34%), a finding that aligns with the previous observation that EA-L-LC exhibits greater sensitivity to pH fluctuations in stability assays. This phenomenon may be attributed to the conformational differences between the two chitosan types. Specifically, LM-CS, with its shorter molecular chains, exhibits greater pH sensitivity in its deprotonation behavior, whereas MM-CS, due to its longer chains and higher degree of entanglement, tends to maintain a more stable coating structure under pH changes. Consequently, EA-L-LC demonstrates a higher release efficiency under SIF conditions. These findings suggest that molecular weight not only determines the initial coating architecture but also influences the stability of chitosan under different environmental conditions [24].

In summary, acting as a robust physical barrier, the chitosan coating effectively suppresses the premature release of EA from liposomes within the simulated gastric environment. This protective effect ensures subsequent sustained release and absorption in the intestinal tract, thereby significantly enhancing the digestive stability of EA.

### 3.8. Chemical Antioxidant Activity

**Figure 8:**
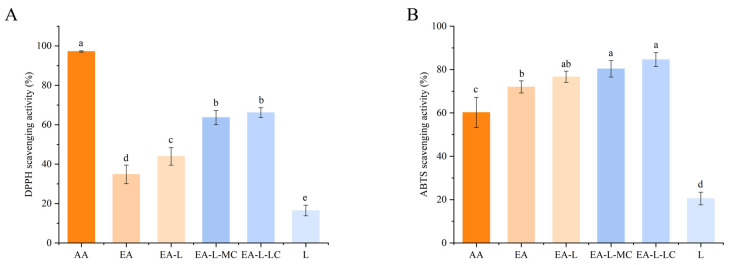
DPPH scavenging capacity (**A**) and ABTS scavenging capacity (**B**) (*n* = 3). Different small letters indicate significant differences (*p* < 0.05).

As illustrated in Figure 8, the encapsulation within liposomes significantly augmented the antioxidant activity of EA compared to its free form. The DPPH radical scavenging capacity of EA-L increased to 43.94% from the 34.81% observed for free EA, while its ABTS radical scavenging activity rose from 71.99% to 76.70%. This enhancement may be attributed to the dual functionality of the liposomal carrier, which not only preserves the intrinsic antioxidant potency of EA but also possesses inherent antioxidant properties of its own [47].

Upon encapsulation with chitosan, the DPPH radical scavenging activities of EA-L-MC and EA-L-LC reached 63.79% and 66.17%, representing significant increments of 1.45-fold and 1.61-fold, respectively, compared to EA-L (43.93%, *p* < 0.05). Similarly, their ABTS radical scavenging capacities improved by 4.83% (EA-L-MC) and 10.38% (EA-L-LC) relative to the 76.70% observed for EA-L. These findings indicate that the chitosan coating further bolsters the antioxidant potency of the liposomes. This enhancement is likely attributable to the presence of amino and hydroxyl groups within the chitosan chains, which act as hydrogen donors to neutralize free radicals [48]. The antioxidant activity of EA-L-LC was slightly higher than that of EA-L-MC, which may be attributed to the differences in molecular weight and degree of deacetylation of chitosan. Generally, chitosan with a lower molecular weight exhibits higher solubility, which facilitates the exposure and reaction of its active amino and hydroxyl groups with free radicals. Furthermore, the degree of deacetylation also influences the antioxidant capacity of chitosan; a higher weight and degree of deacetylation indicate a greater number of free amino groups along the polymer chain, thereby contributing to enhanced antioxidant activity [49].

### 3.9. In Vitro Inhibition of Tyrosinase Activity

**Figure 9:**
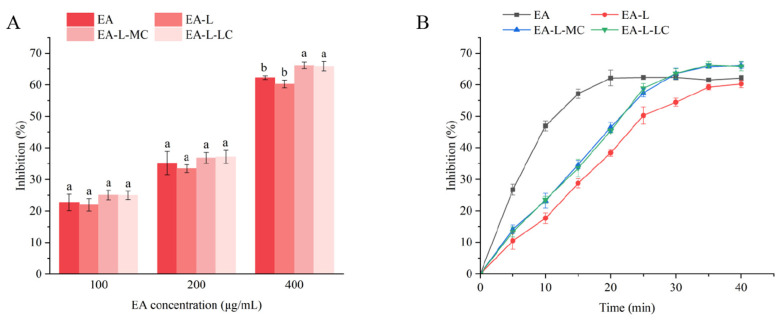
Tyrosinase inhibition activity of EA and liposomes (EA-L, EA-L-MC, EA-L-LC) at different concentrations (**A**) and time course of tyrosinase inhibition at 400 μg/mL (**B**) (*n* = 3). Different small letters indicate significant differences (*p* < 0.05).

Tyrosinase serves as the key rate-limiting enzyme in the melanogenesis pathway, and its inhibition represents a cornerstone strategy for skin whitening and the clinical management of hyperpigmentation disorders [50]. In this study, an in vitro enzymatic assay was employed to systematically evaluate the inhibitory efficacy of free EA, EA-L, and chitosan-modified liposomes (EA-L-LC and EA-L-MC) against tyrosinase activity across a range of concentrations. The results are presented in Figure 9.

Both free and encapsulated EA exhibited a pronounced dose-dependent inhibitory effect on tyrosinase activity. Within the concentration range of 100 to 400 μg/mL, the inhibition activity increased progressively with rising EA concentrations. Specifically, at a concentration of 400 μg/mL, free EA reached a tyrosinase inhibition activity of 62.24%, which was significantly higher than that observed in the low- and medium-concentration groups. This finding is consistent with the established characteristics of EA as a competitive inhibitor of tyrosinase.

At the equivalent concentration of EA, the tyrosinase inhibition activity of EA-L did not differ significantly from that of free EA. These results suggest that within a cell-free enzyme system, encapsulation does not fundamentally alter the inhibitory potency of EA against tyrosinase. Compared to free ellagic acid and EA-L, chitosan-modified ellagic acid liposomes (EA-L-MC and EA-L-LC) exhibited enhanced inhibitory effects on tyrosinase. At concentrations of 100, 200, and 400 μg/mL, the inhibition activity reached 25.03%, 36.81% and 66.12% for EA-L-MC and 24.94%, 37.14% and 65.85% for EA-L-LC, representing increases of 14.19%, 3.39%, and 5.90% and 13.78%, 3.72%, and 5.63% over EA-L, respectively. The IC50 value of chitosan-modified EA liposomes decreased to 261.0 μg/mL (EA-L-MC) and 261.2 μg/mL (EA-L-LC), compared to 287.2 μg/mL for EA and 305.0 μg/mL for EA-L. This phenomenon may be attributed to the intrinsic biological activity of chitosan; prior studies have demonstrated that chitosan exerts a direct inhibitory effect on tyrosinase [51]. The incorporation of chitosan likely yields a synergistic effect within the system.

As illustrated in Figure 9B, at a concentration of 400 μg/mL, the tyrosinase inhibition activity of free EA rose rapidly during the initial stage of the reaction (0–10 min) before leveling off. After 20 min, the inhibition activity eventually stabilized at approximately 62%. The inhibition activity of encapsulated EA increased more slowly than that of free EA. This delayed response is likely because the EA is trapped within the liposomal bilayers, which limits their direct contact with the enzyme. These results suggest that the liposomes provide a sustained-release effect [52]. Compared with the rapid inhibition of free ellagic acid, the slower but sustained inhibitory activity of liposomes and chitosan-modified liposomes against tyrosinase offers superior advantages and practical application significance.

## 4. Conclusions

In this study, EA liposomes were prepared using the thin-film hydration–ultrasonication method, with formulation and processing parameters optimized via RSM. The results indicated that the encapsulation efficiency reached 85.93 ± 0.57% under the optimal conditions of a hydration temperature of 50 °C, 10 min of ultrasonication at 120 W, a 1:22 drug-to-lipid ratio, a 67 min hydration time, and a 5:1 lecithin-to-cholesterol ratio. Building on these findings, low-molecular-weight and medium-molecular-weight chitosans were used for surface modification, successfully producing chitosan-coated liposomes (EA-L-LC and EA-L-MC). Following modification, the encapsulation efficiency increased to 94.72 ± 0.84% and 94.57 ± 1.04%.

TEM and Zeta-potential analyses confirmed successful chitosan deposition, characterized by an increased particle size and a distinctive charge reversal from negative to positive. FTIR spectroscopy further elucidated that the encapsulation and coating processes were governed by hydrogen bonding and electrostatic interactions. Stability assays revealed that chitosan-modified liposomes possessed superior particle size stability, higher EA retention over 30 days (4 °C), and enhanced resistance to acid-induced aggregation (pH = 2.0). In vitro digestion models demonstrated that the chitosan coating facilitated sustained EA release and improved gastrointestinal stability.

The evaluation of antioxidant activity revealed that chitosan-modified EA liposomes exhibited higher scavenging capacities against DPPH and ABTS radicals compared to both unmodified liposomes and free EA. Furthermore, liposomes modified with low-molecular-weight chitosan showed slightly superior antioxidant activity. Regarding the tyrosinase inhibition assays, free EA demonstrated a dose-dependent inhibitory effect, reaching an inhibition percentage of 62.24% at a concentration of 400 μg/mL. At equivalent concentrations, no significant difference was observed between the tyrosinase inhibition activity of free EA and that of the EA liposomes. Surface modification with chitosan led to an increase in the inhibitory activity of the liposomes, which can be attributed to the inherent inhibitory effect of chitosan itself on tyrosinase.

In conclusion, chitosan-modified liposomes represent a robust functional delivery platform with high encapsulation efficiency and stability, offering significant potential for applications in the food and pharmaceutical industries. Furthermore, comparing the two types of chitosan-modified liposomes, EA liposomes modified with low-molecular-weight chitosan exhibited a smaller particle size and higher antioxidant activity, whereas those modified with medium-molecular-weight chitosan demonstrated better stability. Accordingly, different types of chitosan can be selected according to specific requirements.

## Figures and Tables

**Table 1:** The formula of in vitro simulation digestion fluids.

	Component	SGF	SIF
	CaCl_2_ (H_2_O_2_)	10.00 mM	40.00 mM
Electrolyte solution	KH_2_PO_4_	3.50 mM	80.00 mM
	NaCl	120.00 mM	150.00 mM
	MgCl_2_·6H_2_O	3.60 mM	
	KCl	6.00 mM	
	Porcine bile salt		10.00 mg/mL
Enzyme	Pepsin	3.20 mg/mL	
	Trypsin		35.00 mg/mL

**Table 2:** Response surface experiment results.

Number	Factor			EE (%)
	(A) Drug-to-Lecithin Ratio	(B) Hydration Time (min)	(C) Lecithin-to-Cholesterol Ratio	
1	1:20	90	6:1	80.33
2	1:20	60	5:1	85.62
3	1:20	60	5:1	85.21
4	1:20	30	4:1	77.27
5	1:20	60	5:1	84.65
6	1:15	60	6:1	77.84
7	1:15	60	4:1	73.56
8	1:25	30	5:1	79.73
9	1:20	30	6:1	79.09
10	1:20	60	5:1	84.86
11	1:15	30	5:1	74.49
12	1:25	90	5:1	82.70
13	1:20	90	4:1	80.60
14	1:25	60	4:1	81.12
15	1:25	60	6:1	81.20
16	1:20	60	5:1	85.84
17	1:15	90	5:1	77.74

**Table 3:** Results of ANOVA.

Source	Sum of Squares	df	Mean Square	F-Value	*p*-Value	
Model	227.84	9	25.32	74.15	<0.0001	Significant
A	55.76	1	55.76	163.32	<0.0001	**
B	14.55	1	14.55	42.63	0.0003	**
C	4.37	1	4.37	12.79	0.0090	*
AB	0.0196	1	0.0196	0.0574	0.8175	
AC	4.41	1	4.41	12.92	0.0088	**
BC	1.09	1	1.09	3.20	0.1168	
A^2^	58.64	1	58.64	171.75	<0.0001	**
B^2^	33.94	1	34.94	99.42	<0.0001	**
C^2^	39.79.	1	39.79	116.56	<0.0001	**
Residual	2.39	7	0.3414			
Lack of Fit	1.39	3	0.4640	1.86	0.2770	Not significant
Pure Error	0.9977	4	0.2494			
Cor Total	230.23	16				

Note: *R*^2^ = 0.9896, ${R\;}_{adj}^{2}$ = 0.9763. ** *p* < 0.01; * *p* < 0.05.

**Table 4:** Average particle size, polydispersity index, and zeta-potential of different liposomes.

	MPS (nm)	PDI	Zeta-Potential (mV)	EE (%)
L	76.10 ± 3.11d	0.13 ± 0.02a	−47.07 ± 0.85c	-
EA-L	113.73 ± 7.90c	0.11 ± 0.02a	−35.87 ± 0.21b	85.93 ± 0.57b
EA-L-MC	250.83 ± 7.02a	0.10 ± 0.01b	66.23 ± 1.69a	94.57 ± 1.04a
EA-L-LC	219.27 ± 1.80b	0.10 ± 0.01b	63.87 ± 4.46a	94.72 ± 0.84a

L: Blank liposomes; EA-L: EA-loaded liposomes; EA-L-MC: EA liposomes modified with MM-CS; EA-L-LC: EA liposomes modified with LM-CS (*n* = 3). Different letters in the same column indicate significant differences (*p* < 0.05).

## Data Availability

The original contributions presented in this study are included in the article. Further inquiries can be directed to the corresponding author.
